# Stable isotopes reveal the importance of seabirds and marine foods in the diet of St Kilda field mice

**DOI:** 10.1038/s41598-020-62672-x

**Published:** 2020-04-08

**Authors:** Anthony W. J. Bicknell, Benjamin W. Walker, Tom Black, Jason Newton, Josephine M. Pemberton, Richard Luxmoore, Richard Inger, Stephen C. Votier

**Affiliations:** 1 Environment & Sustainability Institute, College for Life and Environmental Sciences, University of Exeter, Cornwall Campus, Penryn, Cornwall TR10 9EZ UK; 2 Institute of Evolutionary Biology, School of Biological Sciences, University of Edinburgh, Edinburgh, EH9 3FL UK; 3 NERC Life Sciences Mass Spectrometry Facility, SUERC, East Kilbride, G75 0QF UK; 4 The National Trust for Scotland, Hermiston Quay, 5 Cultins Road, Edinburgh, EH11 4DF UK; 5 Environmental Biology, College for Life and Environmental Sciences, University of Exeter, Hatherly Building, Prince of Wales Road, Exeter, EX4 4PS UK

**Keywords:** Conservation biology, Conservation biology, Conservation biology, Conservation biology, Conservation biology

## Abstract

Introduced mammals have devastated island nesting seabird populations worldwide. Declines in breeding seabirds on St Kilda, UK, have been linked to climate change and predation from great skuas *Stercorarius skuas*, but the introduced St Kilda field mouse *Apodemus sylvaticus hirtensis* may also play a role by feeding on adults, chicks or eggs. Here, we use stable isotopes in St Kilda mouse blood and potential dietary items to investigate their foraging ecology, specifically focussing on the importance of seabirds and marine foods in their diet. Mice were seasonally sampled at three sites on Hirta, St Kilda over three consecutive years (2010–2012). The δ^13^C and δ^15^N ratios were used in analyses, including isotope niche and dietary source mixing models, to examine foraging behaviour among locations and between seabird breeding seasons. Mice sampled in Carn Mor – where the majority of the island’s seabirds nest - had consistently higher δ^13^C than other locations throughout the year, with δ^15^N also being significantly higher for all but one comparison. The isotopic niche width (SEAs) of Carn Mor mice in each season were distinct from the other locations, and became smaller during the seabird breeding season. Dietary mixing models revealed that seabirds made up a large proportion of the diet for mice from Carn Mor, particularly during the seabird breeding season. In conclusion, our work reveals that seabird-derived foods are likely to form a significant part of the diet of St Kilda mice populations located in and around breeding colonies. It is unclear however, whether this is from scavenging or predation of seabirds, or through their discarded food items. Given that mice have had significant effects on seabird populations elsewhere, it is important to carry out further work to determine whether mice are a significant cause of seabird mortality in this island ecosystem.

## Introduction

Human colonisation of islands has caused profound environmental transformations, extinction events, and cascading ecosystem changes^1–4^. Many of the negative impacts of human colonisation can be attributed to the introduction of non-native species, which often accompanied human arrival, and subsequently became invasive^5–7^. The introduction of invasive species is a leading cause of extinctions worldwide^8^, with effects ranging from direct predation and competition^1,9,10^, to indirect impacts, such as vegetation change, breeding site destruction, and altered nutrient availability^7,11–13^.

Introduced mammals have had some of the most damaging effects on island wildlife, altering trophic relationships and leading to large numbers of extinctions^1,5,14^. Seabirds have been particularly severely impacted by introduced mammals. This is typically because seabirds have evolved in the absence of mammalian predators and because of their bet-hedging life history characteristics^15,16^. For instance, introduced rats (*Rattus sp*) have negatively affected at least 75 species of seabirds across 10 families^17^ and overall, introduced cats (*Felis sp*) and rats are some of the biggest drivers of global seabird declines^5,10,14,17–19^. However, mice (*Apodemus & Mus spp*) may also represent a previously largely overlooked threat^5^. For instance, following removal of rats and cats, mice will predate seabird eggs and chicks to such an extent as to cause significant population declines^9,20–24^. Seabird populations are threatened globally^25^, and invasive species such as mice are one of the top three threats^26^, therefore, understanding their impact on seabirds is a conservation priority.

**Figure 1:**
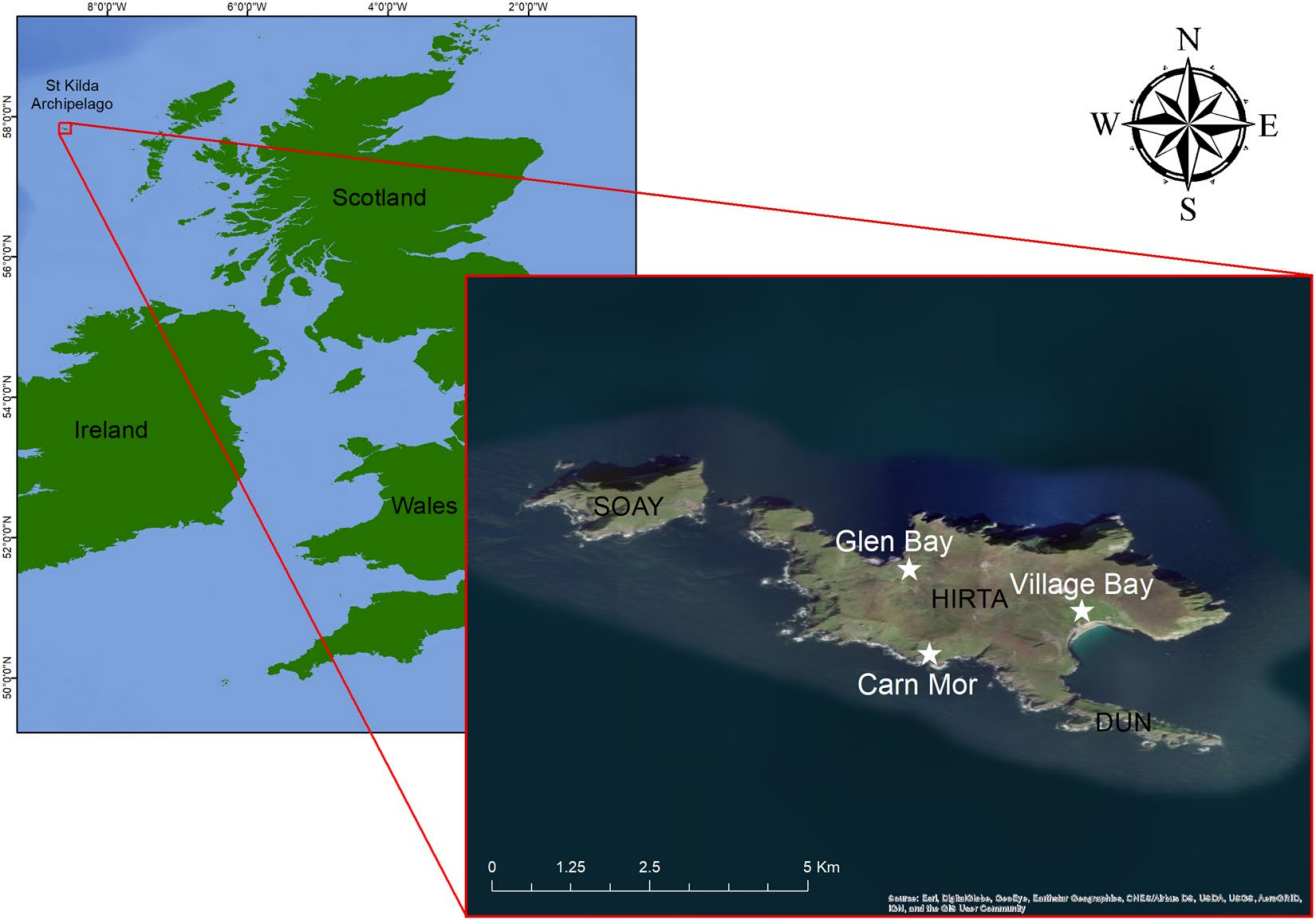
The St Kilda archipelago of Hirta, Soay and Dun (fourth island - Boreray - not shown). The three locations (Glen Bay, Carn Mor and Village Bay) where St Kilda mice where caught and sampled are indicated. (ArcGIS Online basemap image. Sources: Esri, DigitalGlobe, GeoEye, i-cubed, USDA FSA, USGS, AEX, Getmapping, Aerogrid, IGN, IGP, swisstopo, and the GIS User Community).

The St Kilda archipelago (Fig. 1) hosts the largest seabird breeding assemblage in the north-east Atlantic, with internationally important populations of northern fulmar (*Fulmarus glacialis*), Leach’s storm-petrel (*Oceanodroma leucorhoa*), European storm-petrel (*Hydrobates pelagicus*), Atlantic puffin (*Fratercula arctica*), Manx shearwater (*Puffinus puffinus*), common guillemot (*Uria aalge*) and razorbill (*Alca torda*) (Table 1)^27–29^. St Kilda is also home to an endemic field mouse *Apodemus sylvaticus hirtensis*^30^, which is assumed to have been introduced by man, potentially over a thousand years ago^31^. A sub-species of house mouse *Mus musculus muralis* also evolved on the islands^32^, but became extinct soon after the human evacuation in 1931, leaving the field mouse as the only land predator. Seabirds and mice have co-existed since their arrival; however burrow-nesting Leach’s storm-petrel have declined by 54% over the period 1999–2003^33^. While this decline is thought to be largely due to predation by great skuas (*Stercorarius skua*)^33–35^, there is circumstantial evidence of predation by St Kilda field mice that could have contributed to this decline^27^.

**Table 1:** Seabird species with populations >1000 breeding individuals on St Kilda. Species marked with an asterisk breed on the islands of Hirta and Dun and are therefore overlap spatially with the St Kilda mouse. All data from JNCC Seabird 2000 census^28^.

Seabird Species	Breeding individuals
Kittiwake Rissa tridactyla*	7,772
Northern fulmar Fulmarus glacialis*	136,869
Manx shearwater Puffinus puffinus*	9,606
European storm-petrel Hydrobates pelagicus*	2,242
Leach’s storm-petrel Oceanodroma leucorhoa*	90,866
Common guillemot Uria aalge*	31,347
Razorbill Alca torda*	3,378
Northern gannet *Morus bassanus*	119,244
Atlantic puffin Fratercula arctica*	284,528

Here, we use carbon (δ^13^C) and nitrogen (δ^15^N) stable isotope (SI) data to understand more about the little-known foraging ecology of the St Kilda field mouse. Stable isotope analyses (SIA) are commonly used to infer diet and trophic relationships, providing powerful tools to understand foraging ecology, particularly for species like mice that have cryptic lifestyles^36^. The isotopic composition of sampled tissues reflects feeding during the period of tissue synthesis^37,38^. In coastal environments, δ^13^C is useful for differentiating between animals feeding in locations where terrestrial (C3 plants) or marine (phytoplankton) primary production is at the base of the food web, due to the latter’s photosynthetic pathway producing relatively higher δ^13^C values that are represented in consumers tissues^39–41^. The δ^15^N values are helpful for elucidating trophic ecology by virtue of the stepwise increase of ~3–4‰ for each trophic level^39,42,43^, and tend to be higher in marine environment due to longer food chains than in terrestrial systems. When these isotopes are used in combination, values can reveal important information on isotopic niche width (an approximation of the trophic niche), with greater widths often used to indicate a broader niche width^44^, and identify dietary proportions when putative food sources have been isotopically characterised (SI mixing models)^45^. We collected mouse blood and potential diet items to be included in SIA from three locations on Hirta, the largest island in the St Kilda archipelago (Fig. 1); Carn Mor, Glen Bay and Village Bay. These sites varied in their likely food availability: Village and Glen Bay were likely to be dominated by terrestrial vegetation, invertebrates and Soay sheep (*Ovis aries*) carrion; while mice in Carn Mor had additional access to large numbers of breeding seabirds between April and September, notably the second largest Leach’s storm-petrel colony in Europe^28^. If seabirds are an important food for St Kilda mice, we predict individuals from Carn Mor will have isotopic values with strong a marine signature from seabirds and their food (i.e. elevated δ^13^C values)^39,41^ and be different from other areas on the island^46^. We also predict that the seasonal availability of seabird material (which peaks during seabird breeding but  becomes scarce at other times of the year) would lead to changes in isotopic niche width and dietary composition, particularly in Carn Mor mice.

## Methods

### Study area

Samples were collected from live trapped St Kilda mice, at Carn Mor, Glen Bay, Village Bay (Fig. 1). Trapping locations were geographically distinct from one another (i.e. they were separated by escarpments or habitat unsuitable for mice to occupy) and were representative of the area of the island in which they were placed. At each site, trapping was focused on areas with extensive rock (natural or built into walls) since a pilot study captured few mice in open areas.

Carn Mor (Fig. 1, 57°48′34″N 8°36′6″W) is located on the west of the island and consists of extensive steep sloped talus fields (60–70% cover), with areas of scattered short grass, leading to the edge of high sea cliffs. It is the nesting site for large numbers of northern fulmar, Leach’s storm-petrel, Manx shearwater, and Atlantic puffin (total breeding adults across all species >20,000), as well as smaller numbers of European storm-petrel and common guillemot^28,29^. The main seabird breeding season is between April and September, after which birds depart the island until the following year.

Village Bay (Fig. 1, 57°48′53″N 8°34′15″W) is located on the sheltered southeast corner and is the main bay on the island. The area largely consists of short, mixed grasses and herbs with areas of heather on higher slopes. Village Bay was previously permanently inhabited by humans, and as such contains stone walls, cleits (stone storage or drying huts) and abandoned houses, where small numbers of fulmar and European storm-petrel nest in the summer (<100 breeding adults). This area also contains the highest density of feral Soay sheep on the island due to the high quality of vegetation found there, with sheep carcasses being present in the area resulting from overwinter mortality. This is the only sampling site close to the manned Ministry of Defence (MoD) base, and buildings occupied seasonally by National Trust for Scotland and research staff^47^.

Glen Bay (Fig. 1, 57°49′10″N 8°36′16″W) is in the north coast of the island and consists of steep grass slopes interspersed with talus at one edge, leading to a flat area of mostly grassland. Areas of talus are used by a small number of fulmars, Manx shearwaters, and great skuas for nesting (<100 breeding adults). The bay is quite exposed, and during December and January receives no direct sunlight. During these winter months, and often extending to March, this area commonly contains fresh sheep carcasses.

### Mouse sampling

Eleven trapping sessions took place between March 2010 and September 2012 in spring (March), early summer (June), late summer (August) and late autumn (November). Each session consisted of five consecutive nights of trap deployment at each site. Sites were trapped sequentially as far as logistically possible; for example, it was not considered safe to trap at Carn Mor during strong westerly winds.

Longworth traps (Penlon Ltd., Oxford, UK) were arranged at each site in a grid of 100 trapping stations (arranged in 10 × 10 grid format, 10 m from each other). Each station contained two traps, totalling 200 traps per grid. Traps were wrapped in bubble wrap to provide insulation and were baited with shelled peanuts, and carrot pieces, and stuffed with dry grass as bedding. Traps were placed within the cover of rocks or walls where possible, before being left in the same location for the duration of the trapping session. Trap position was repeatable between sessions. The traps were checked each morning for mice.

Newly caught animals were marked either with a passive integrated transponder (PIT) tag (AVID Plc, Lewes, UK), inserted into the scruff of the neck, or with individual ear punch patterns. Biometric data, including sex, age class, reproductive and body condition, were recorded for all caught mice, when weather permitted^47^. On the first capture per trapping session for each mouse, a ~100 µl blood sample was taken through a submandibular venepuncture. Mice were then released at the point of capture, and traps reset with fresh bait and bedding. All work was subject to the University of Edinburgh’s ethical review panel, and experimental protocols were undertaken under licence from the UK Home Office. All procedures were performed in accordance with relevant guidelines and regulations for working with live vertebrates.

### Sample processing

Blood samples were drawn into non-heparinised capillaries. Only red blood cells (RBC) were processed (as below) and used in subsequent data analysis, which due to the turnover rate of RBC, would allow us an indication of mouse diet over 1–3 weeks^48–51^.

A range of potential food items found at the sample sites were also opportunistically sampled. These included invertebrates (beetles, spiders, earwigs, woodlice, worms, slugs, bristletails); sheep carrion; plants (Caryophyllales, Fabales, Lamiales, Paoles, Asterales); fungi; and egg, blood and muscle samples from seabirds (Atlantic puffin, Leach’s storm-petrel and northern fulmar). Seabird and sheep carrion samples were lipid extracted to remove any confounding influence of these compounds^52^. All samples were reduced to dry weight in an incubator at 60 °C for 48 hours. Samples were then homogenised using a pestle and mortar in preparation for isotope analysis.

Stable isotope analysis was undertaken at the Natural Environment Research Council Life Science Mass Spectrometry Facility in East Kilbride, Scotland, UK, using continuous-flow isotope mass spectrometry. Samples were analysed using a Flash HT elemental analyser, with a Thermo Electro Delta XP isotope ratio mass spectrometer (IRMS). Stable isotope data are reported in delta notation (δ), where δ^15^N and δ^13^C = ((R_sample_/R_Standard_) − 1) × 1000, R = ^15^N/^14^N or ^13^C/^12^C. Delta values are expressed per thousand (‰) relative to the ratio of international reference standards of atmospheric nitrogen and Vienna PeeDee Belemnite (VPDB) for carbon.

### Data analysis

We first used general linear models (GLMs) to test how δ^15^N and δ^13^C of mouse RBC were affected by sample site (three levels = Carn Mor, Glen Bay, Village Bay), month (four levels = March, June, September, November), and year (three levels = 2010, 2011, 2012). Interaction terms between month and site were included in addition to main effects of site, month and year. Normality of the residuals, and homoscedasticity were checked for both models, and δ^13^C values were found to be skewed. A constant of +35‰ was applied to the δ^13^C data to produce positive values in order to use the Box-Cox function in the ‘MASS’ package^53^. δ^13^C values were square root transformed in line with recommended Box-Cox transformations (λ = 0.5), which resulted in a largely normal distribution, with some non-linearity around the tails.

#### Dietary prey/food analysis

To investigate whether δ^15^N and δ^13^C prey group values varied between the seabird breeding season and non-breeding season we used MANOVA and ANOVA tests. Normality of the residuals and homoscedasticity were checked for all models. A constant of +10‰ and +35‰ was applied to δ^15^N and δ^13^C data, respectively, to produce positive values for Box-Cox transformation (using most likely λ value) when residuals where not normally distributed. We further investigated whether δ^15^N and δ^13^C prey group values varied between sampling site using non-parametric Kruskal-Wallis rank sum and post-hoc Wilcoxon pairwise test, due to non-normal residuals and/or lack of homoscedasticity for MANOVA and ANOVA models. The non-parametric test results are reported but (interestingly) the significant differences were found to be the same as the excluded parametric models.

#### Isotopic niche width analysis

The isotopic niche width was calculated using standard ellipse areas (SEA) in SIBER^44^ (requiring the use of ‘rjags’^54^ and ‘hdrcde’^55^). This is a bivariate measure of the distribution of individuals in isotopic space, with the ellipses set to enclose 95% of the data^44^. A Bayesian estimate of SEA (SEA_*B*_) was calculated using Markov Chain Monte Carlo (MCMC) simulation (50,000 iterations) to produce modes and 95% credible intervals (CrI). Differences in the size of isotopic niche widths (as SEA_*B*_) among locations and between seabird breeding seasons were evaluated by calculating the probability that the relative posterior distributions of the niche size were significantly smaller or larger (α = 0.05). The extent of isotopic niche width overlap (%) between locations and seasons was calculated from SEA based on the maximum likelihood fitted ellipses.

#### Dietary source bayesian mixing models

Bayesian mixing models within SIMMR^56^ were used to predict the relative proportions of putative dietary sources consumed by mice from each sampling location and between seabird breeding and non-breeding seasons. Food item SI values were combined into 5 dietary source groups (invertebrates, plants, sheep, fungi and seabirds) and split into the seabird season they were collected in (breeding and non-breeding). Key to mixing model efficacy is an accurate characterization of potential sources. This was checked by observing whether mixture values (mice RBC) lay within the fuzzy convex hull of the sources values in an iso-space plot^45^.

During the process of assimilating dietary nutrients, isotopic ratios in the consumer change relative to the dietary values, termed ‘trophic discrimination factors’ (TDF). These factors need to be incorporated into mixing models to enable correct proportioning of dietary sources. Since species- or tissue- specific TDFs were unavailable for St Kilda field mouse we used published TDFs from blood in a feeding experiment on laboratory mice (Δ^13^C = 1 ±SD 0.15‰; Δ^15^N = 3 ±SD 0.2‰)^57^. Concentration dependence means and standard deviations for model dietary source groups were derived from the % weight of carbon and nitrogen in the putative food items.

The models were based on MCMC simulation methods (iterations = 50,000, burn in = 5,000, thinning = 10, chains = 4) and run separately for samples collected in seabird breeding (BS) and non-breeding (NBS) seasons. Model convergence and fit were checked using Gelman diagnostic values and plotting the posterior predictive distributions.

All analyses were conducted using R version 3.6.2^58^.

## Results

### Sampling mice and potential diet

Of 591 individual mice caught, we obtained blood from 588, from which we measured δ^13^C and δ^15^N. Across the three sites, 339 potential diet samples were collected covering a range of possible food items from 16 different Orders and 6 Phyla.

### δ^15^N in mouse blood

**Figure 2:**
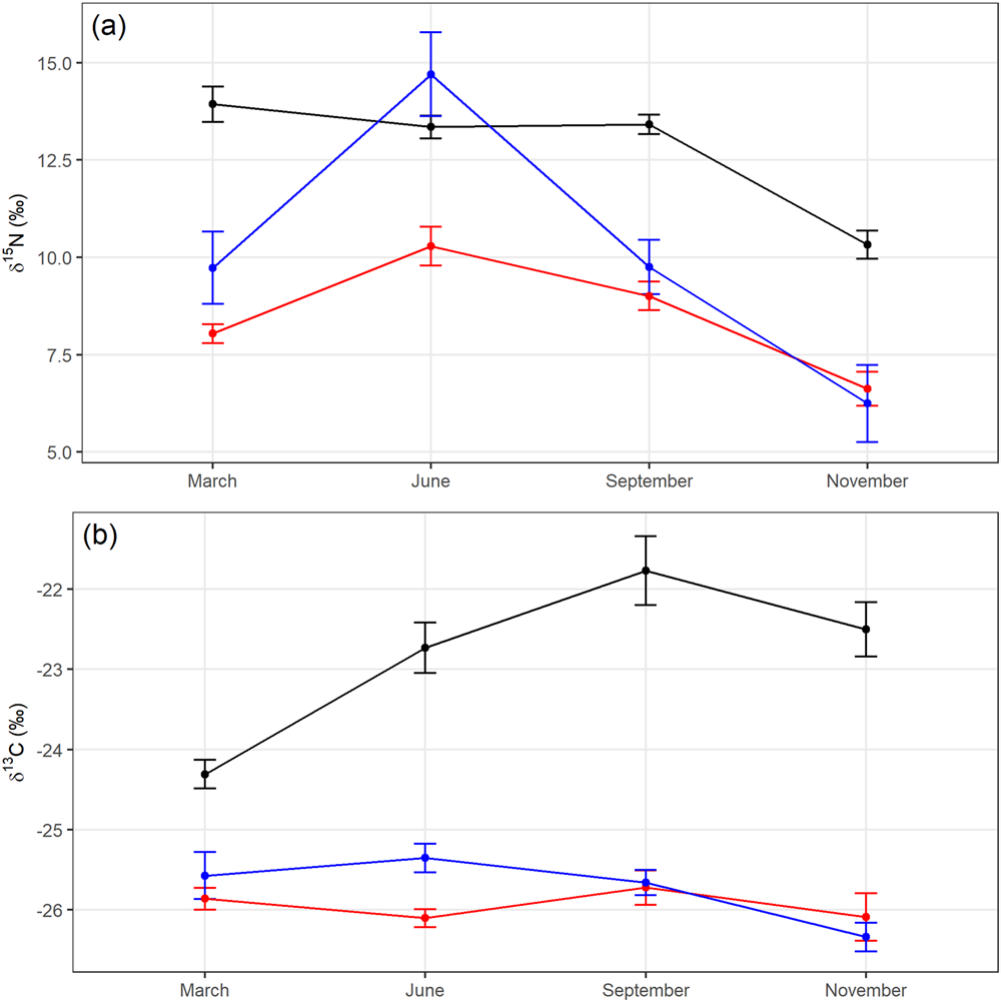
δ^15^N and δ^13^C values in mouse red blood cells (mean ± 95% CI) at three study locations on Hirta, St Kilda (black = Carn Mor, red = Glen Bay, blue = Village Bay) in four sampling months.

δ^15^N values ranged from 3.6‰ to 19.1‰ (mean ± SD = 10.72 ± 3.13‰), and differed significantly among sites on Hirta (*F*_*2,573*_ = 313.532, *p* = <0.001; Fig. 2a). Mice from Village Bay (mean = 9.99‰, 95% CI: 9.37, 10.63) and Glen Bay (mean = 8.55‰, 95% CI: 8.29, 8.81) were significantly lower than those at Carn Mor (mean = 12.61‰, 95% CI: 12.36, 12.87), but were not significantly different from each other.

δ^15^N values varied significantly by sampling month within sites (*F*_*6,573*_ = 27.006, *p* = <0.001, Fig. 2a). Values at Carn Mor for March, June and September did not differ significantly from each other, but all three were significantly higher than November. δ^15^N values for March at Glen Bay were significantly different than in all other months (Fig. 2a, Supplementary Table 1). Values at Glen Bay for June and September did not differ from each other, while values for November were significantly lower than all others (Fig. 2a, Supplementary Table 1). At Village Bay, δ^15^N values did not differ between March and September, but values in June were higher than all other months, while values in November were lower (Fig. 2a, Supplementary Table 1). Sample year had no significant effect on δ^15^N (*F*_*2,562*_ = 0.279, *p* = 0.756).

### δ^13^C in mouse blood

δ^13^C values ranged from −27.09‰ to −19.17‰ (mean ± SD = −24.51 ± 1.90‰), and differed significantly by site (*F*_*2,573*_ = 814.616, *p* = <0.001), and by month on Hirta (*F*_*3,573*_ = 26.633, *p* = <0.001, Fig. 2b). Mice in Carn Mor (mean = −22.83‰, 95% CI: −23.03, −22.64) had significantly higher δ^13^C values than those from Glen Bay (mean = −25.93‰, 95% CI: −26.03, −25.84) and Village Bay (mean = −25.73‰, 95% CI: −25.84, −25.61), which did not significantly differ from each other.

δ^13^C values varied by sampling month within sites (*F*_*6,573*_ = 24.673, *p* = <0.001, Fig. 2b). Values in Carn Mor for March were significantly lower than values from June, September and November (Fig. 2b, Supplementary Table 1). In Glen Bay, δ^13^C values did not significantly differ for all four months (Fig. 2b, Supplementary Table 1). At Village Bay, δ^13^C values did not significantly differ for the first three months, while November had significantly lower values than these months (Fig. 2b, Supplementary Table 1). Sample year had no influence on δ^13^C (*F*_*2,566*_ = 0.889, *p* = 0.412).

### Isotopic space and niche width

**Figure 3:**
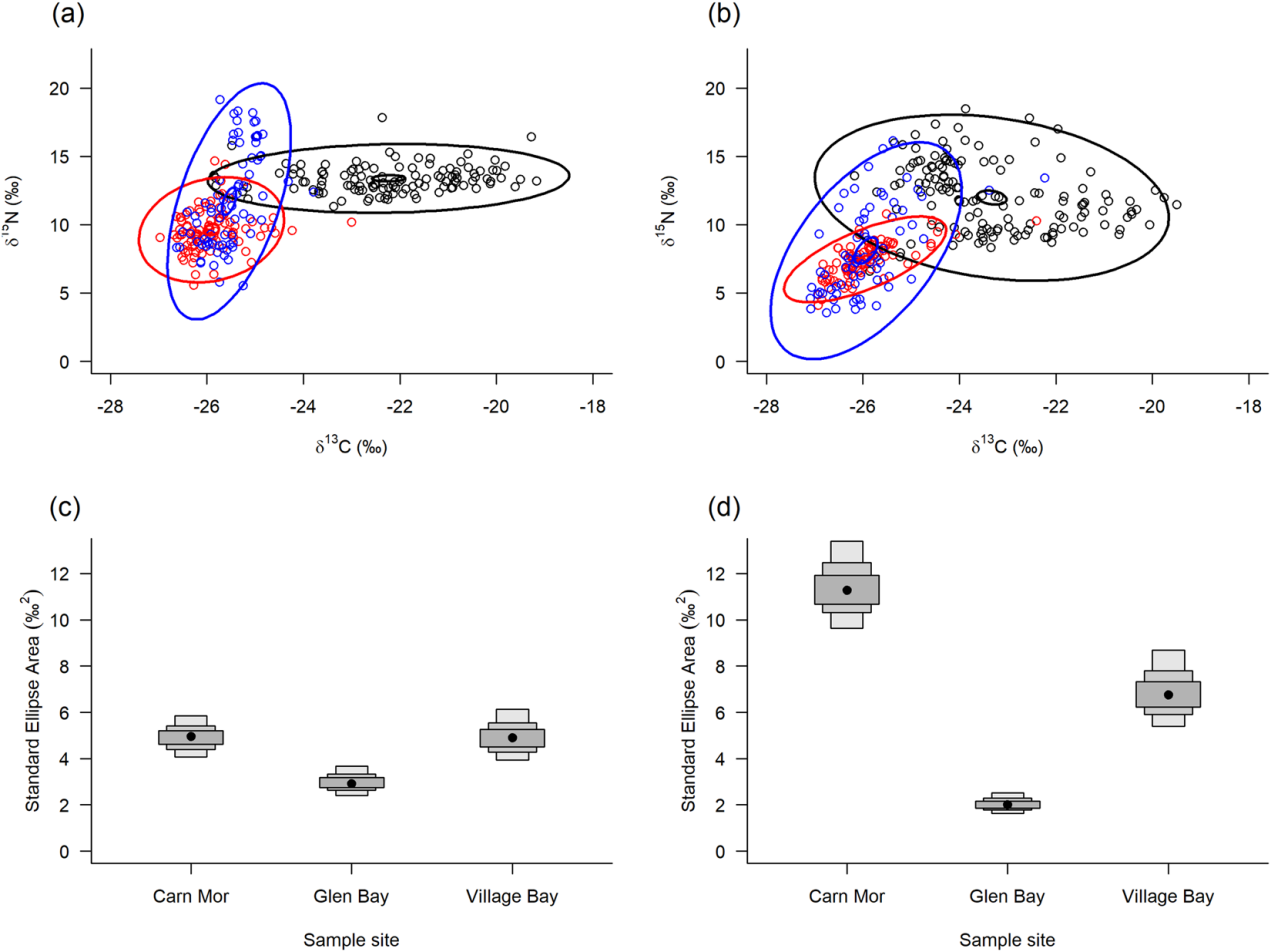
Bayesian ellipse plots (δ^15^N and δ^13^C) and associated standard ellipse areas (SEA_*B*_) for St Kilda mice RBC values during seabird breeding (**a**,**c**) and non-breeding (**b**,**d**) seasons. Ellipses: black = Carn Mor, red = Glen Bay, blue= Village Bay, large = 95% prediction ellipse and small = 95% confidence interval of the bivariate mean. SEA_*B*_ plots (**c**,**d**) show modes, 95% and 99% credible intervals.

The Bayesian ellipse plots and SEA_*B*_ values revealed isotopic niche width differences among locations and between seabird breeding seasons (Fig. 3a–d).

#### Among locations

Mice in Carn Mor occupy a different isotopic space (iso-space) compared to mice in Village Bay and Glen Bay, being largely driven by higher δ^13^C values (Fig. 3a,b). The proportion ellipse overlap between Carn Mor and Glen Bay or Village Bay was small during the breeding season (Carn Mor and Glen Bay = 4.3%, Carn Mor and Village Bay = 8.8%), compared to between Glen Bay and Village Bay (42.8%) (Fig. 3a). The proportion was similar in the non-breeding season between Carn Mor and Glen Bay (5.4%), but increased considerably between Carn Mor and Village Bay in the non-breeding season (19.7%). Although the ellipse overlap between Glen Bay and Village Bay decreased in the non-breeding season (29.4%), the iso-space of Glen Bay was entirely contained with the Village Bay iso-space (Fig. 3b).

SEA_*B*_ values (isotopic niche width) were greater for Carn Mor and Village Bay compared to Glen Bay (probability > 0.99), but not between each other (P = 0.48 & 0.52) during the breeding season (Fig. 3c). During the non-breeding season the SEA_*B*_ was greater in Carn Mor than both Village Bay and Glen Bay (P > 0.99), and Village Bay was greater than Glen Bay (P = 1) (Fig. 3d).

#### Between seabird breeding seasons within locations

**Figure 4:**
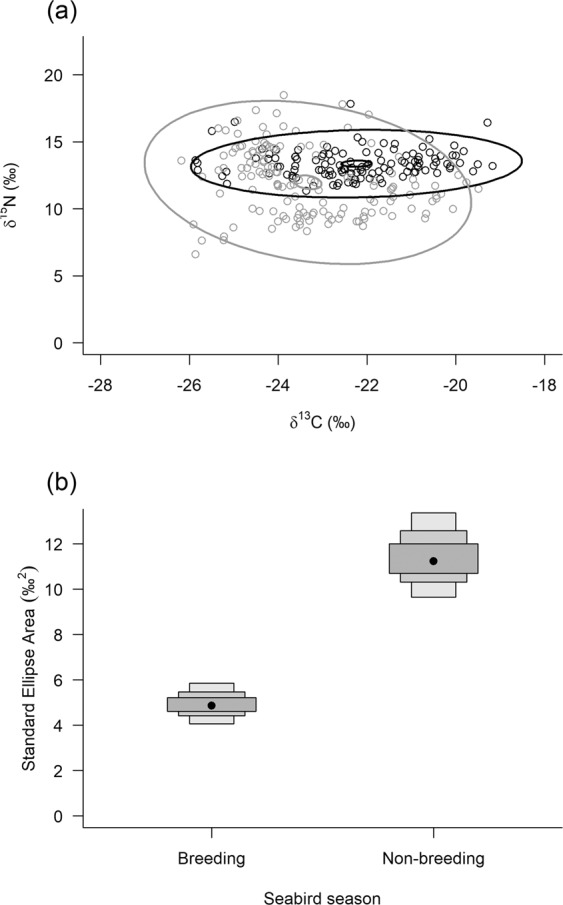
Bayesian ellipse plots (**a**; δ^15^N and δ^13^C) and associated standard ellipse areas (**b**; SEA_*B*_) for Carn Mor mice RBC values during seabird breeding and non-breeding seasons. Ellipses: black = breeding, grey = non-breeding, large = 95% prediction ellipse and small = 95% confidence interval of the bivariate mean. SEA_*B*_ plots (**b**) show modes, 95% and 99% credible intervals.

The proportion ellipse overlap between breeding seasons was least for Carn Mor mice (Carn Mor = 33.3%, Glen Bay = 39.9%, Village Bay = 46.6%), and SEA_*B*_ was greater in the non-breeding season (P = 1) (Fig. 4). This was the same for Village Bay (>0.98; Supplementary Fig. 2), but the SEA_*B*_ for Glen Bay was greater during the breeding season (Supplementary Fig. 1).

### Dietary prey/food and proportions

#### Dietary prey/food

The δ^15^N and δ^13^C values did not differ for fungi or seabirds between sample sites (Kruskal-Wallis tests, all *p* > 0.05; Supplementary Tables 2, 3 and Fig. 3). Village Bay had lower δ13C and δ15N values for plants and invertebrates, and higher δ13C and lower δ15N for sheep, compared to the other two sites (All *p* < 0.05; Supplementary Tables 2, 3 and Fig. 3). Carn Mor and Glen Bay are similar in all prey types, except δ15N values for sheep (*p* < 0.05; Supplementary Table 3 and Fig. 3).

#### Dietary proportions

**Figure 5:**
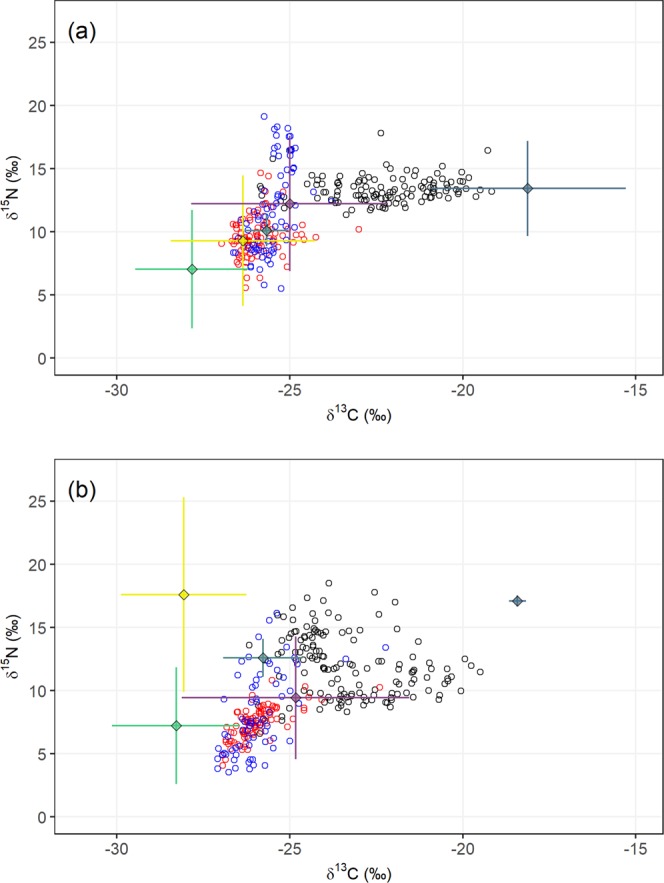
δ^15^N and δ^13^C bivariate plots of individual St Kilda mice RBC values (circles) and the dietary sources (means ± SD) used in the Bayesian isotope mixing models (SIMMR) for the seabird breeding (**a**) and non-breeding (**b**) seasons. Circles: black = Carn Mor, red = Glen Bay and blue = Village Bay. Dietary sources: yellow = fungi, green = plants, light blue = sheep, grey = seabirds, and purple = invertebrates.

**Figure 6:**
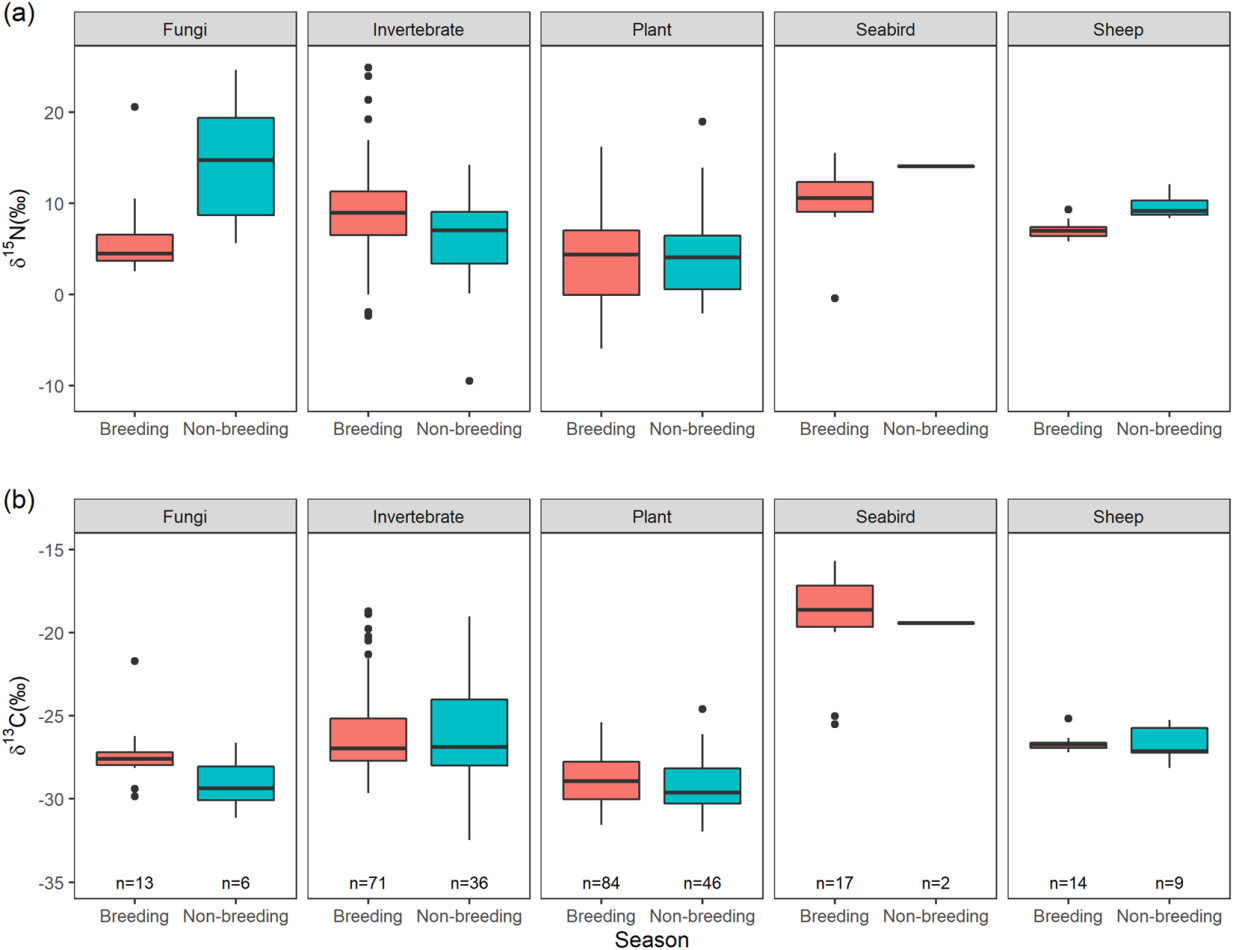
δ^15^N (**a**) and δ^13^C (**b**) values of dietary sources during seabird breeding and non-breeding seasons used in SIMMR isotope mixing models.

The RBC SI values (mixtures) fitted well within fuzzy convex hulls of the dietary source values, indicating good characterisation of potential sources for the seasonal models (Fig. 5). The δ^13^C values did not differ for any dietary source between the breeding and non-breeding season (MANOVA tests *p* > 0.05), but δ^15^N was significantly higher for fungi and sheep, and lower for invertebrates during the non-breeding season (MANOVA tests *p* < 0.01; Fig. 6). Model convergence and fit were good for each season and location.

**Figure 7:**
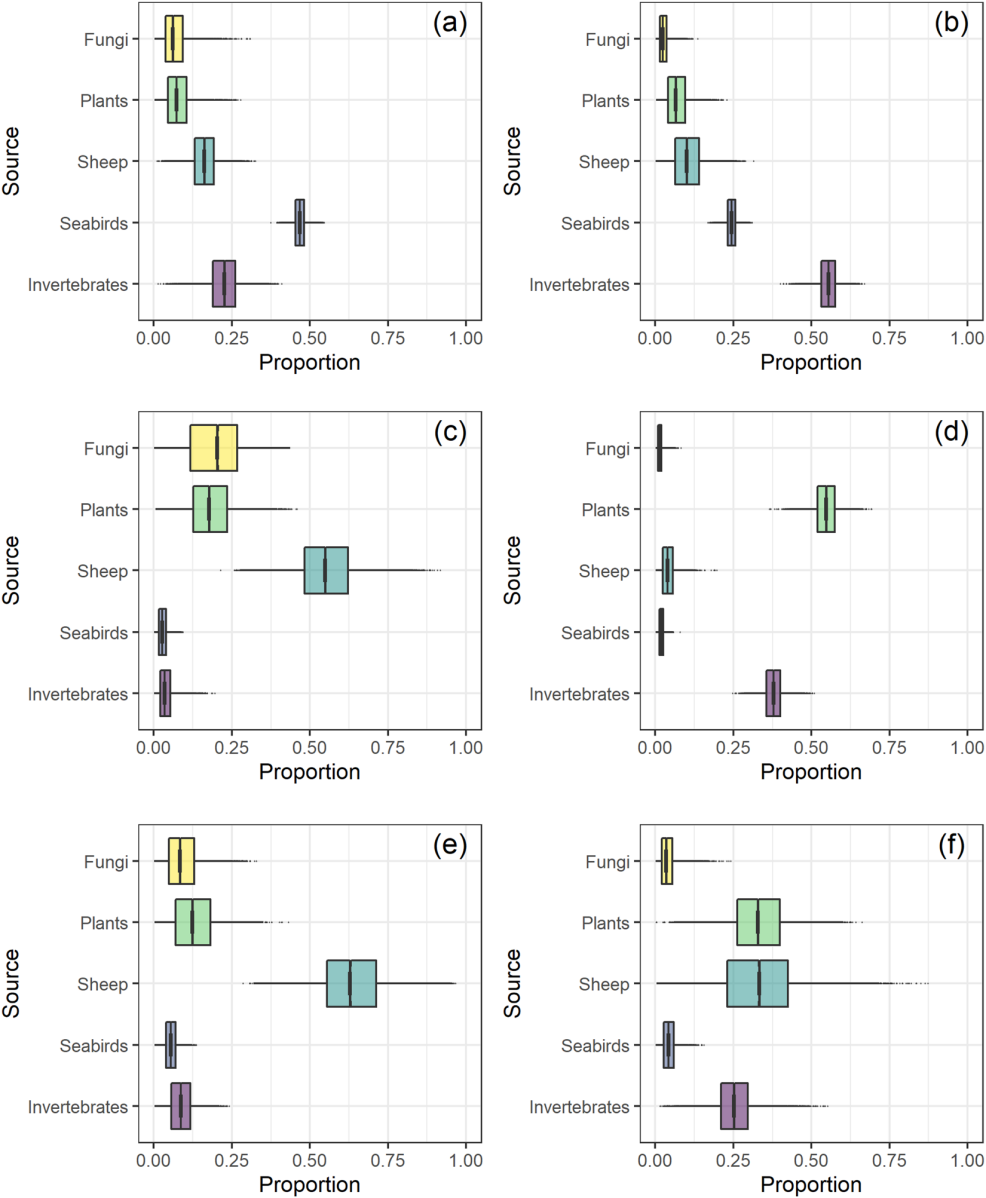
Bayesian mixing models predicted relative proportions (mean and 50% credible intervals of posterior distribution) of putative dietary sources consumed by mice from Carn Mor (**a**,**b**), Glen Bay (**c**,**d**) and Village Bay (**e**,**f**) in seabird breeding seasons (breeding season = a,c,e;  non-breeding season = b,d,f).

The predicted relative dietary proportions (*Prp*) consumed by mice varied among locations and changed between seasons (Fig. 7). During the breeding season, seabirds were predicted to make up the largest proportion of the diet for Carn Mor mice (*Prp* = 0.46 ± 0.02 SD; Fig. 7a), but sheep were the largest for Glen Bay (*Prp* = 0.55 ± 0.1 SD; Fig. 7c) and Village Bay (*Prp* = 0.64 ± 0.11 SD; Fig. 7e) mice. During the non-breeding season, invertebrates were predicted to be consumed most by Carn Mor mice (*Prp* = 0.46 ± 0.02 SD), with seabirds contributing the second largest proportion (*Prp* = 0.24 ± 0.02 SD; Fig. 7b). For Glen Bay, plants (*Prp* = 0.55 ± 0.04 SD) and invertebrates (*Prp* = 0.38 ± 0.03 SD; Fig. 7d) were the largest proportion, and Village Bay mice were predicted to feed on plants (*Prp* = 0.33 ± 0.09 SD), sheep (*Prp* = 0.33 ± 0.14 SD) and invertebrates (*Prp* = 0.26 ± 0.07 SD; Fig. 7f). Seabirds were predicted to contribute <5% of the diet in Glen Bay and Village Bay mice during both seasons.

## Discussion

Our study reveals that δ^15^N and δ^13^C values, isotopic niche width and diet composition of St Kilda mice vary among sub-populations on the main island (Hirta), and between seabird breeding and non-breeding seasons. Mice in Carn Mor had generally higher δ^13^C and δ^15^N values compared to the other two sites, their isotopic niche width changed considerably when seabirds were breeding on the island, and this was reflected in a large portion of their diet predicted to consist of seabird-derived food (Fig. 7). By contrast, mice from Glen Bay and Village Bay had consistently lower δ^13^C values, much smaller changes in isotopic niche width between seabird breeding seasons (Supplementary Figs. 1 & 2) and <5% of their diet was predicted to be from seabird derived food (Fig. 7). This is in line with our expectations that if mice were feeding on seabirds it would be most prevalent among mice living sympatrically with large numbers of breeding seabirds in Carn Mor during April to September. The implications of these findings for understanding the impacts of mice on seabird communities on St Kilda, and in general, are discussed below.

### Carn Mor mouse sub-population

The most likely explanation for the elevated δ^15^N and δ^13^C values of mice in Carn Mor compared with Village Bay and Glen Bay, is the consumption of seabird-derived foods^24,59^. It is possible that nutrients from seabird guano may have enriched the Carn Mor ecosystem and thus elevated the isotopic values of prey items that occur there (i.e. plants, invertebrates, fungi, sheep), however, δ^15^N or δ^13^C values in food items were not disproportionately different between Carn Mor and the other locations (Supplementary Tables 2, 3 and Fig. 3). The prey data actually suggests the Village Bay area may have variation in carbon and nitrogen input for certain food items but this was not consistent across taxonomic groups. The consistently higher δ^15^N and δ^13^C values in Carn Mor mice relative to other locations, therefore, are likely a result of differences in diet (i.e. a higher proportion of seabird-derived food at Carn Mor) rather than underlying base nutrient input.

The higher δ^13^C values are indicative of marine derived food^39–41^ and the higher δ^15^N values suggest feeding at a higher trophic level^42^. The latter is indicative of the longer food chains in marine systems that would be reflected in seabird derived food. Consumption of marine food from coastal habitats, such as crustaceans and algae, would also lead to similarly high δ^13^C values, but this seems extremely unlikely since the slopes of Carn Mor sit atop vertical sea cliffs, too high for mice to climb safely (>40 m). However, we cannot entirely discount the possibility that mice feed on fish and zooplankton dropped or discarded by adult seabirds on return from foraging trips.

There were also strong seasonal differences in mice from Carn Mor (Fig. 4). The separation in isotopic space between breeding and non-breeding is illustrated by the different orientation and size of the SIBER model ellipses (Fig. 4a). The breeding season ellipse falls largely within the non-breeding season range, with a narrower range of δ^15^N values. The narrow δ^15^N range contributes to a small isotopic niche width in the breeding season (Fig. 4b). The isotopic niche width is much greater during the seabird non-breeding season (Fig. 4b), which we suggest is a result of mice being forced to seek other food sources. Mixing models predicted a switch from a mainly seabird derived food diet (>40%) during the seabird breeding season, to one dominated by invertebrates (>50%). Correspondingly, the large range of species ( >10 species), and associated SI values (Fig. 6), within the invertebrate dietary group aligns with the increase in isotopic niche width found for the Carn Mor mice during the seabird non-breeding season.

These predictions corroborate the claims of Bicknell *et al*.^27^ that St Kilda mice either scavenge or possibly predate some seabirds (particularly smaller species like Leach’s storm-petrel). Moreover, in the winter months when few seabirds remain at Carn Mor, the relative contribution of seabird prey dropped steeply, although not completely. During these months a small number of seabird may still be present around the island but, more importantly, remains and remnants from the recent breeding season (e.g. adult or chick seabird remains or unviable/failed eggs) will still be available for consumption and may represent a significant portion of their diet. The decreasing δ^13^C values in Carn Mor mice from November to March (Fig. 3) also suggest the seabird derived food resource diminishes, or becomes depleted, over the winter months (non-breeding period), supporting this hypothesis. While more work is required to determine the method by which seabird derived food is obtained by mice (i.e. whether it is scavenging or more predatory in nature), it is clear that mice in this part of St Kilda are making considerable use of this food source.

### Glen Bay and Village Bay sub-populations

The Glen Bay and Village Bay mice had much lower δ^15^N and δ^13^C values throughout the year, except for a dramatic increase in δ^15^N for Village Bay mice in June (Fig. 2). The increase suggests an input of higher trophic level food items, possibly from Soay sheep (for instance, May is the end of the lambing period) and/or from people working on or visiting the island (there are more visitors during summer). The human resident population (National Trust for Scotland and Ministry of Defence personnel) are accommodated in Village Bay and visitors also spend most time in this part of the island. Although sheep carrion was predicted as the main food source for both Village Bay and Glen Bay sub-populations during the period incorporating June (BS) (Fig. 7), there are a small number of Village Bay individuals that had δ^15^N values at the top range of dietary sources that could possibly represent individuals feeding on another source not sampled in this study (Fig. 5a). For example, if potential human-derived food contained high δ^15^N values, predictions for the Village Bay sub-population’s diet may be improved if this dietary source was added to the models. The majority of mouse samples, however, were well within model parameters to provide credible results. The isotopic niche width and predicted diet composition for Village Bay mice suggest their diversity of food items (i.e. taxonomic orders) were highest, especially in the winter months.

Glen Bay’s isotopic niche width was narrow and consistent between seasons (Supplementary Fig. 1), although the predicted diet composition changed substantially from sheep carrion, plants and fungi during summer to plants and invertebrates during winter (Fig. 7). Mice in Glen Bay therefore appeared to be somewhat independent of the seasonal changes in seabirds (Carn Mor) and humans/sheep (Village Bay).

### St Kilda field mouse diet and seabird predation

This is the first study on the foraging ecology of St Kilda field mice, providing important information on this endemic subspecies that features in the Statement of Universal Value for this World Heritage Site^60^. It is therefore of interest that mice appear to feed on seabird-derived food, as previously suspected on St Kilda^27^, and documented at other seabird colonies^7,9,20,21,24,59,61^. However, our results reveal nothing about the way in which mice obtain seabird-derived food. There is likely to be a large quantity of carrion available around the seabird colony in the form of lost or deserted eggs, as well as dead adults and chicks, which could be easily scavenged by mice. It is also common to see discarded fish dropped by birds, mostly puffins. Moreover, great skuas frequently only partially consumed carcases and regurgitate pellets of their seabird prey (including large numbers of storm-petrels)^62^, providing another potential source of seabird carrion for mice. However, circumstantial evidence indicates that for very small seabirds like Leach’s storm-petrel, St Kilda mice may take eggs or chicks^27,47,59^ and studies elsewhere show that mice are capable of tackling large live prey such as albatross chicks^20^. Previous studies on the impacts of introduced mice on seabirds have nearly all involved the house mouse (*Mus musculus*). This is the first indication that field (wood) mice (*Apodemus sylvaticus*) also interact with seabirds. More work is needed to better understand the foraging behaviour of St Kilda field mice – camera traps would provide one way to determine whether they are taking seabird prey live or simply as carrion. Without any such additional information, it would be inappropriate to draw firm conclusions about the potential contribution of mice to seabird mortality on Hirta, particularly considering the significance of this endemic mouse subspecies.

To conclude, this study highlights the power of stable isotope analyses to study the diet of cryptic species like mice, and in this instance the hitherto poorly studied endemic St Kilda field mouse. Our results indicate that mice breeding close to a large seabird colony are isotopically distinct from mice elsewhere on the island and that these values varied in line with the temporal availability of seabirds. The evidence is clear that mice on Carn Mor feed on seabirds, although it is unknown whether this is from scavenging carrion or predating live birds, or viable eggs. Further work is required to study the true nature of the interaction between St Kilda field mice and the threatened seabird populations in this small and remote island archipelago.

## Supplementary information

Supplementary information.
