# Supplementary material for: Stable isotopes reveal the importance of seabirds and marine foods in the diet of St Kilda field mice
Source: Sci Rep. 2020 Apr 8;10:6088. doi: 10.1038/s41598-020-62672-x (PMC7142145; doi:10.1038/s41598-020-62672-x)
Supplement: Supplementary file 1 — Supplementary information. [file 41598_2020_62672_MOESM1_ESM.docx]

Stable isotopes reveal the importance of seabirds and marine foods in the diet of St Kilda field mice

Anthony W J Bicknell, Benjamin W Walker, Tom Black, Jason Newton, Josephine M Pemberton, Richard Luxmoore, Richard Inger & Stephen C Votier.

**Supplementary Table 1** δ^15^N and δ^13^C values from St Kilda mice bloods in Carn Mor, Glen Bay and Village Bay. Mean and 95% confidence intervals (CI) for each sampling month (2010-2012).

| **Location** | **Month** | **Mean δ^15^N (‰)** | **95% CI**  **(lower, upper)** | **Mean δ^13^C (‰)** | **95% CI**  **(lower, upper)** |
| --- | --- | --- | --- | --- | --- |
| Carn Mor | March | 13.93 | 13.48, 14.39 | -24.31 | -24.49, -24.13 |
|  | June | 13.35 | 13.06, 13.64 | -22.73 | -23.05, -22.42 |
|  | September | 13.42 | 13.17, 13.67 | -21.77 | -22.20, -21.34 |
|  | November | 10.33 | 9.96, 10.69 | -22.50 | -22.84, -22.16 |
| Glen Bay | March | 8.04 | 7.80, 8.28 | -25.86 | -26.00, -25.73 |
|  | June | 10.29 | 9.75, 10.78 | -26.10 | -26.22, -25.99 |
|  | September | 9.01 | 8.64, 09.38 | -25.72 | -26.39, -25.79 |
|  | November | 6.63 | 6.19, 7.06 | -26.09 | -26.39, -25.79 |
| Village Bay | March | 9.73 | 8.80, 10.66 | -25.57 | -25.87, -25.28 |
|  | June | 14.70 | 13.63, 15.78 | -25.35 | -25.53, -25.18 |
|  | September | 9.75 | 9.05, 10.45 | -25.66 | -25.50, -25.82 |
|  | November | 6.25 | 5.26, 7.24 | -26.34 | -26.52, -26.16 |

**Supplementary Table 2** δ^13^C summary values for prey samples collected in Carn Mor, Glen Bay and Village Bay, with Kruskal-Wallis rank sum test and Wilcoxon pairwise test results. * = significant pairwise difference (*p* < 0.05); SD = standard deviation; IQR = interquartile range

| **Dietary source** | **Location** | **n** | **Mean** | **SD** | **Median** | **IQR** | **Kruskal-Wallis rank sum test**  **(*p*-value)** | **Wilcoxon pairwise tests (<0.05)** | | |
| --- | --- | --- | --- | --- | --- | --- | --- | --- | --- | --- |
| Fungi | Carn Mor | 6 | -28.0 | 1.22 | -27.6 | 1.44 | 0.299 | - | - |  |
|  | Glen Bay | 6 | -26.9 | 2.84 | -27.6 | 1.30 |  | - |  | - |
|  | Village Bay | 7 | -28.7 | 1.41 | -28.1 | 1.81 |  |  | - | - |
| Invertebrate | Carn Mor | 39 | -24.8 | 3.46 | -25.1 | 5.09 | <0.01 | - | * |  |
|  | Glen Bay | 33 | -26.0 | 2.51 | -27.0 | 3.27 |  | - |  | * |
|  | Village Bay | 35 | -27.2 | 1.64 | -27.4 | 1.91 |  |  | * | * |
| Plants | Carn Mor | 42 | -28.4 | 1.75 | -28.2 | 2.32 | <0.01 | - | * |  |
|  | Glen Bay | 40 | -29.0 | 1.73 | -29.1 | 2.55 |  | - |  | * |
|  | Village Bay | 48 | -29.5 | 1.03 | -29.6 | 1.39 |  |  | * | * |
| Seabirds | Carn Mor | 15 | -18.7 | 2.27 | -18.6 | 2.42 | 0.323 | - | - |  |
|  | Glen Bay | 2 | -22.1 | 4.87 | -22.1 | 3.44 |  | - |  | - |
|  | Village Bay | 2 | -19.6 | 0.03 | -19.6 | 0.03 |  |  | - | - |
| Sheep | Carn Mor | 3 | -27.7 | 0.46 | -27.7 | 0.46 | 0.015 | - | * |  |
|  | Glen Bay | 5 | -26.1 | 0.94 | -25.7 | 1.70 |  | - |  | - |
|  | Village Bay | 15 | -26.7 | 0.39 | -26.8 | 0.36 |  |  | * | - |

**Supplementary Table 3** δ^15^N summary values from prey samples collected in Carn Mor, Glen Bay and Village Bay, with Kruskal-Wallis rank sum test and Wilcoxon pairwise test results. * = significant pairwise difference (*p* < 0.05); SD = standard deviation; IQR = interquartile range

| **Dietary source** | **Location** | **n** | **Mean** | **SD** | **Median** | **IQR** | **Kruskal-Wallis rank sum test**  **(*p*-value)** | **Wilcoxon pairwise tests (<0.05)** | | |
| --- | --- | --- | --- | --- | --- | --- | --- | --- | --- | --- |
| Fungi | Carn Mor | 6 | 12.2 | 6.63 | 9.73 | 10.3 | 0.109 | - | - |  |
|  | Glen Bay | 6 | 7.86 | 8.33 | 4.62 | 2.70 |  | - |  | - |
|  | Village Bay | 7 | 6.99 | 5.43 | 4.49 | 5.25 |  |  | - | - |
| Invertebrate | Carn Mor | 39 | 9.65 | 6.03 | 10.0 | 6.27 | <0.001 | - | * |  |
|  | Glen Bay | 33 | 8.67 | 3.24 | 8.69 | 3.36 |  | - |  | * |
|  | Village Bay | 35 | 6.41 | 4.81 | 6.42 | 3.50 |  |  | * | * |
| Plants | Carn Mor | 42 | 5.70 | 4.00 | 6.51 | 5.18 | <0.001 | - | * |  |
|  | Glen Bay | 40 | 4.74 | 4.54 | 4.99 | 6.29 |  | - |  | * |
|  | Village Bay | 48 | 2.19 | 3.81 | 1.93 | 5.03 |  |  | * | * |
| Seabirds | Carn Mor | 15 | 10.4 | 3.65 | 10.6 | 2.99 | 0.221 | - | - |  |
|  | Glen Bay | 2 | 10.8 | 2.64 | 10.8 | 1.87 |  | - |  | - |
|  | Village Bay | 2 | 13.9 | 0.84 | 13.9 | 0.59 |  |  | - | - |
| Sheep | Carn Mor | 3 | 11.0 | 0.93 | 10.6 | 0.87 | <0.001 | * | * |  |
|  | Glen Bay | 5 | 8.91 | 0.46 | 8.78 | 0.79 |  | * |  | * |
|  | Village Bay | 15 | 7.23 | 1.02 | 7.18 | 1.24 |  |  | * | * |


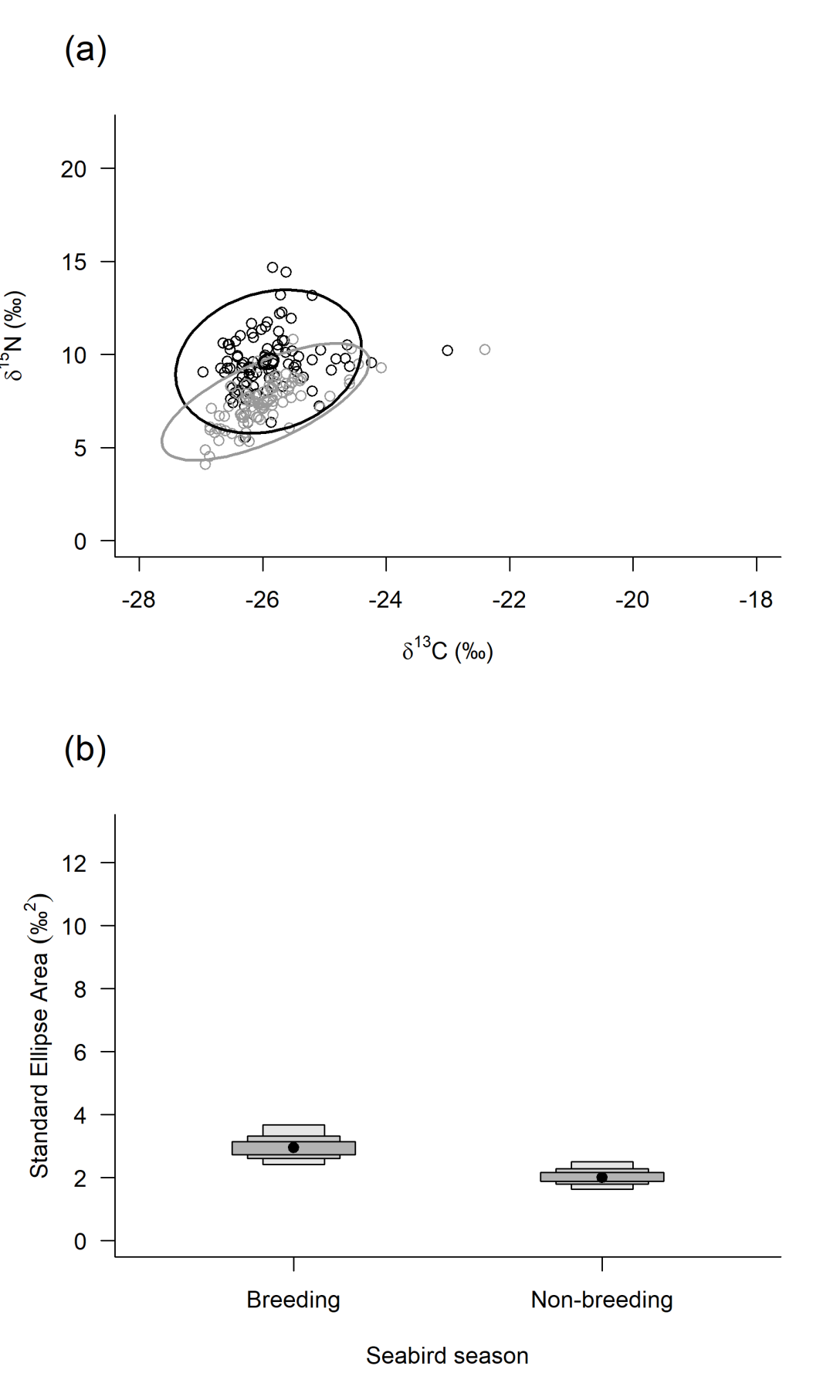


**Supplementary Fig 1** Bayesian ellipse plots (δ^15^N and δ^13^C) and associated standard ellipse areas (SEA*_B_* ) for Glen Bay mice RBC values during seabird breeding and non-breeding seasons. Ellipses: black = breeding, grey = non-breeding, large = 95% prediction ellipse and small = 95% confidence interval of the bivariate mean. SEA*_B_* plots (b) show modes, 95% and 99% credible intervals.

**
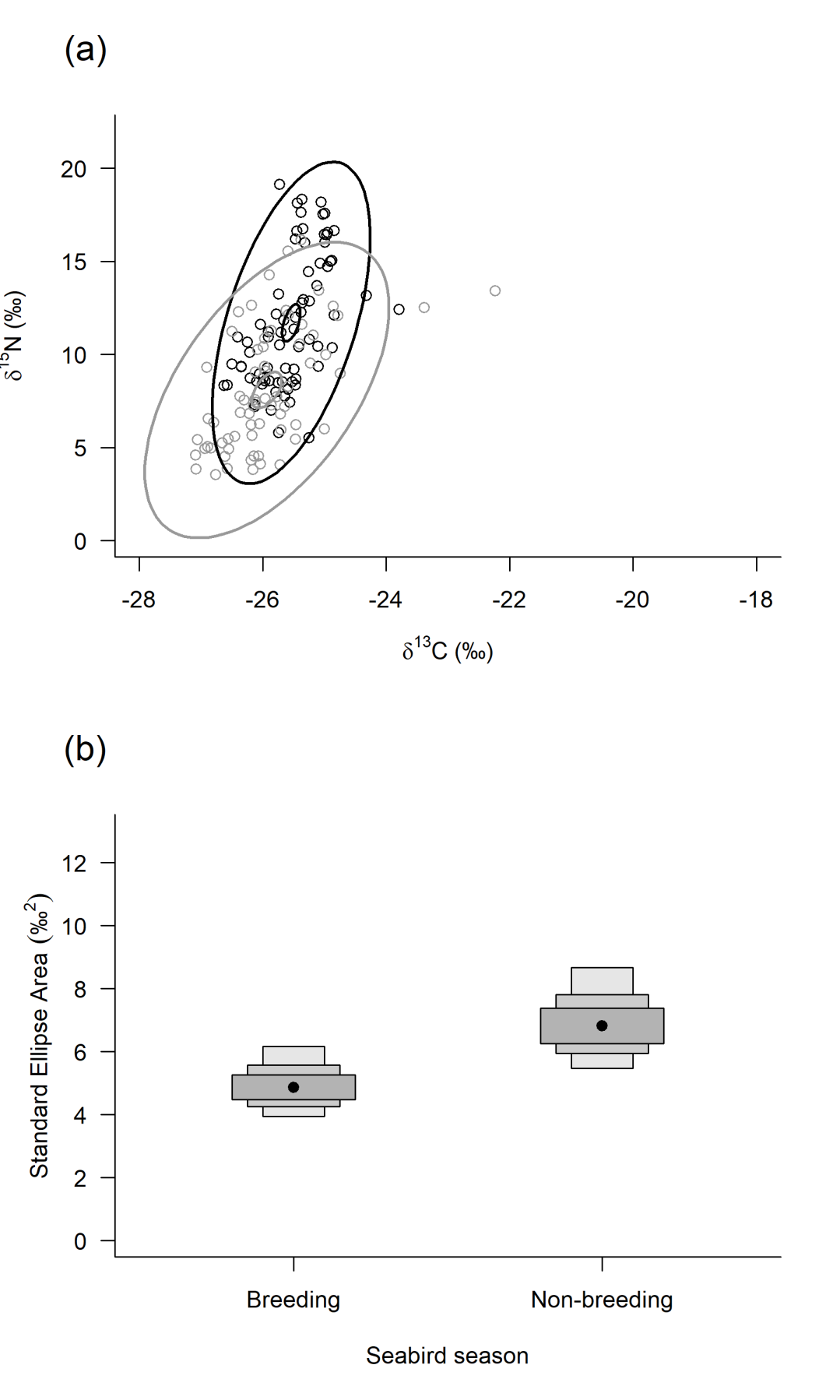
**

**Supplementary Fig 2** Bayesian ellipse plots (δ^15^N and δ^13^C) and associated standard ellipse areas (SEA*_B_* ) for Village Bay mice RBC values during seabird breeding and non-breeding seasons. Ellipses: black = breeding, grey = non-breeding, large = 95% prediction ellipse and small = 95% confidence interval of the bivariate mean. SEA*_B_* plots (b) show modes, 95% and 99% credible intervals.


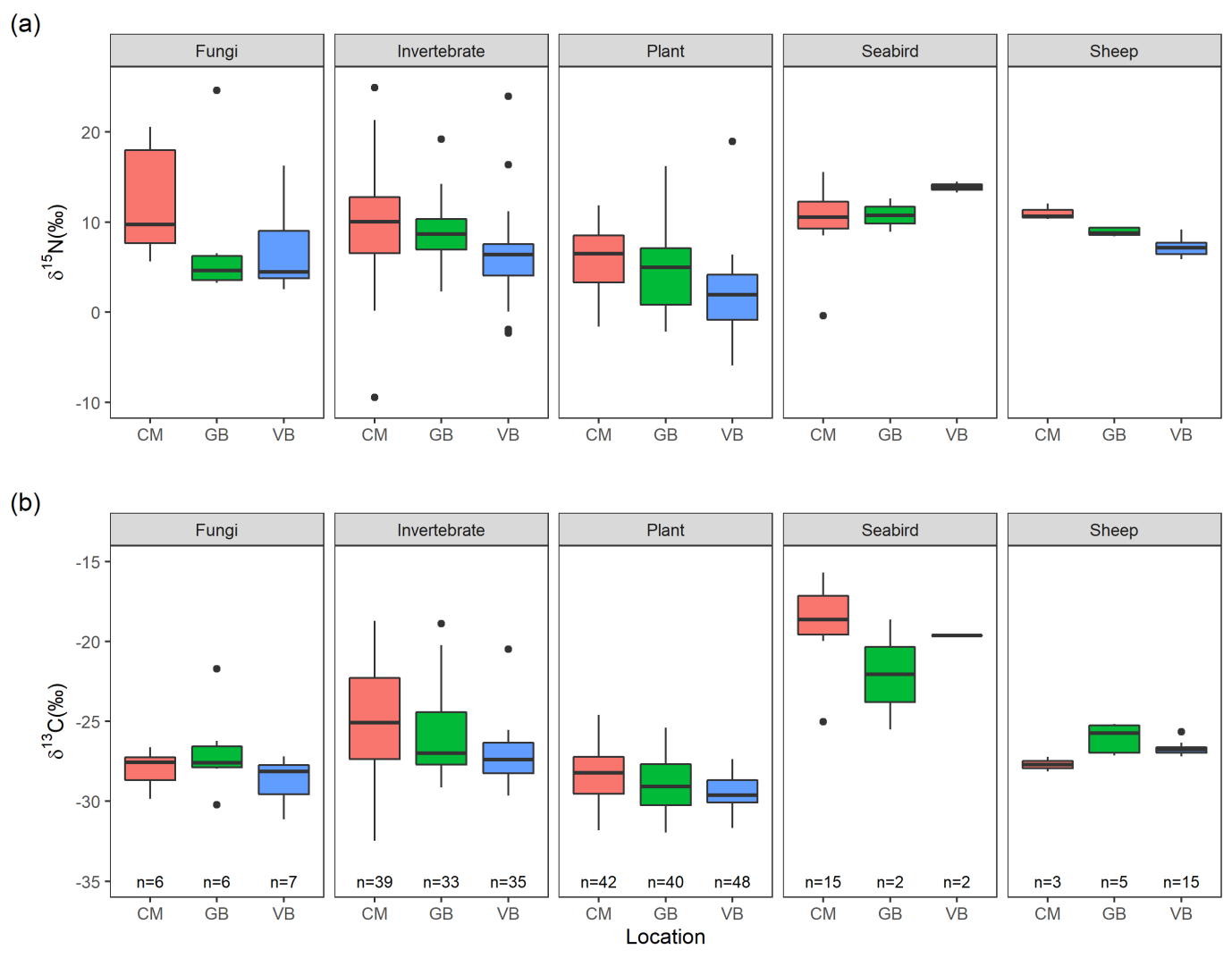


**Supplementary Fig 3** δ^15^N (a) and δ^13^C (b) values of dietary sources in each location (CM = Carn Mor, GB = Glen Bay, VB = Village Bay)
